# Long-term follow-up results of prostate capsule-sparing and nerve-sparing radical cystectomy with neobladder: a single-center retrospective analysis

**DOI:** 10.3389/fruro.2024.1355605

**Published:** 2024-05-21

**Authors:** Zaisheng Zhu, Yiyi Zhu, Hongqi Shi, Penfei Zhou, Yadong Xue, Shengye Hu

**Affiliations:** ^1^ Department of Urology, Jinhua Hospital Affiliated to Zhejiang University School of Medicine, Jinhua, China; ^2^ Department of Endocrinology, Affiliated Second Hospital to Zhejiang University School of Medicine, Hangzhou, China

**Keywords:** bladder cancer, radical cystectomy, prostate capsule sparing, nerve sparing, long-term follow-up

## Abstract

**Objective:**

This study aims to investigate and analyze the feasibility, oncological outcomes, functional efficacy, and complications with the prostatic capsule sparing (PCS) as well as the nerve sparing (NS) in radical cystectomy for bladder cancer.

**Patients and methods:**

Between January 2007 and December 2021, 67 total cystectomies with PCS and 54 with NS were performed at our institution. The inclusion criteria for PCS were as follows: proactive, fully informed patient consent; negative transurethral resection of the bladder neck; normal prostate-specific antigen (PSA) level < 4 ng/dL; and normal transrectal ultrasonography with biopsy of any suspicious nodes. Patients received complete oncological and functional follow-ups. The Kaplan-Meier method was utilized to characterize survival outcomes after surgery.

**Results:**

The median follow-up times for PCS and NS were 144 and 122 months, respectively. Cumulative survival estimated the 5- and 10-years cancer-specific survival were 93.0% and 88.7% for the PCS group and 79.7% and 79.6% for the NS group, respectively (*p* = 0.123). In terms of function, the daytime urinary control at 3, 6, and 12 months postoperatively was 80.60%, 97.01%, and 100% in the PCS group, and 53.70%, 85.19%, and 94.44% in the NS group, respectively (*p* = 0.002, 0.023, and 0.100); and nocturnal urinary control was 62.69%, 94.03%, and 98.51% in the PCS group, and 40.74%, 72.22%, and 87.04% in the NS group, respectively (*p* = 0.016, 0.001, and 0.022). The erectile function recovery revealed that 62.69% and 40.74% of patients returned to preoperative levels (International Index of Erectile Function (IIEF)-5 score ≥ 15) in the PCS and NS groups, respectively (*p* = 0.016). Considering complications within 30 days after surgery, 4.48% and 7.69% patients had Clavien ≥ III complications in the PCS and NS groups, respectively (*p* = 0.700).

**Conclusion:**

The PCS provides better restored urinary control and sexual function than the NS technique and does not affect oncological outcomes. However, PCS is prone to bladder-neck obstruction complications and requires closer long-term follow-up.

## Introduction

Radical cystectomy (RC) and pelvic lymph node dissection (PLND) are the “gold standard” treatments for recurrent high-risk non-muscle invasive and muscle-invasive bladder cancer. Nonetheless, complications, including urinary incontinence and sexual dysfunction, occur easily and require abdominal wall stoma or neobladder replacement (1, 2), which seriously affects the quality of life. Several nerve and urethral sphincter preserving surgeries have been performed to improve function. Among them, two major types of nerve-preserving surgeries existed, including prostate capsule sparing (PCS) total cystectomy, first proposed by Schilling in 1990 (3), and nerve-sparing (NS) cystoprostatectomy, first reported by Schlegel in 1987 (4). Because uroepithelial carcinomas are highly malignant and fatal, physicians prioritize the oncological outcome and radical surgery. Techniques that preserve function (e.g., nerve-sparing) are less frequently used (2, 5–9). Consequently, the follow-up data on long-term postoperative oncological (survival) and functional recovery outcomes remain limited. Recently, nerve-sparing surgeries have increased with the advancement of robotics, other devices, and surgical techniques (1, 5, 10–12). Herein, we retrospectively analyzed cases with PCS and NS techniques over 15 years at our institution and obtained longer-term follow-up cases to report their postoperative oncological and functional outcomes.

## Clinical information and methods

### Study population

From January 2007 to December 2021, 497 men underwent RC plus urinary diversion at our hospital. The orthotopic neobladder was performed in 157 cases, of which 36 cases had received non-nerve sparing surgery preoperatively due to sexual inactivity or erectile dysfunction or those who did not require preservation of sexual function. The remaining 121 cases were included in the study, of which 30 received conventional open surgery, and 91 underwent minimally invasive laparoscopic surgery. The PCS technique was employed in 67 cases, of which 24 underwent conventional open surgery and 43 received laparoscopic minimally invasive surgery. The NS surgery was performed in 54 cases, of which 6 were conventional open and 48 were laparoscopic minimally invasive. Patients who were more sexually active before surgery [this aspect was asked of every patient during preoperative counselling, and erection function is assessed using the IIEF-5 questionnaire (13, 14)] and who were adamant about preserving sexual function were selected for function–preserving cystectomy (PCS or NS). Patients were offered PCS only when they met the following inclusion criteria: (1) patients who requested sexual function preservation (preoperative International Index of Erectile Function (IIEF)-5 score (13, 14) of the erectile function were ≥ 15) and gave fully informed consent; (2) bladder cancer cT1-T3 N0 M0 stages, no tumor in the bladder neck verified by cystoscopy or biopsy; (3) normal PSA levels (< 4 ng/dL), transrectal ultrasound, and MRI, or when there was a suspicious prostate nodule, a negative puncture biopsy was performed. NS was performed on the remaining patients who did not meet these conditions.

## Surgical techniques

### The technique of prostate capsule-sparing

Open PCS technique included bilateral PLND, identification of the vas deferens, bladder, and seminal vesicles and their posterior separation, as well as the prostatic bladder junction, and treatment of the prostatic envelope using the Millin method. The prostate adenoma was removed from its peritoneal capsule and resected along with the bladder. Frozen sectioning was performed on the urethra of the prostate section and the distal ureters bilaterally specimens. Simultaneously, a new bladder was constructed, a ureter was implanted, and anastomosed to the prostate envelope capsule. This group included 24 cases.

### Laparoscopic PCS technique

The laparoscopic PCS technique included bilateral PLND, identification and preservation of the vas deferens, posterior separation of the bladder from the seminal vesicles, and identification and dissection of the prostatic bladder junction to complete cystectomy. The bladder neck was then closed with a continuous suture or a clamped Foley balloon to prevent spillage of tumor cells. The prostate adenoma was then removed from its capsule and assigned with the specimen for frozen sectioning. Simultaneously, a neobladder construct was constructed *in vitro* (outside the abdominal cavity), and a ureter was implanted. The neobladder was anastomosed to the prostate envelope laparoscopically. Forty-three cases were enrolled in this group.

## NS technology

### Open NS

Open NS included bilateral PLND, identification of the vas deferens, posterior separation of the bladder from the seminal vesicles, and the prostate tip. The prostate section was processed using the classical method of preserving the neurovascular bundle (neurovascular bundle) (4). The specimen of total prostate and bladder dissection was then subjected to pathologic examination along with the distal ureter. A neobladder was constructed, and the ureter was implanted and anastomosed to the urethral tip. There were six cases in this group.

### Laparoscopic NS

Laparoscopic NS included bilateral PLND, identification of the vas deferens, posterior separation of the bladder from the seminal vesicles, and apical prostate section. The prostate section was handled using a nerve-sparing approach of total intra-fascial radical prostatectomy (15). The total prostate and bladder specimens were then pathologically examined along with the distal ureter. The neobladder was constructed *in vitro* (outside the abdominal cavity), and the ureter was implanted. The neobladder was anastomosed laparoscopically to the urethral tip. A full description of this technique was previously published (16). Forty-eight cases were performed in our group.

All neobladder constructs were performed *in vitro* (outside the abdominal cavity), using W-shaped, U-shaped, modified Studer-shaped [we performed a modified anti-reflux ureteral neobladder anastomosis (17)] of detubulated ileum and U-shaped detubulated sigmoid colon.

### Covariates and outcome evaluation

Tumors were staged and classified according to the 2002 TNM classification. Postoperative follow-up was conducted as follows: every three months in the first year, six months in the second year, and annually in the third year. The follow-up included a physical examination, blood routine, blood biochemistry, PSA, urine routine, chest X-ray, urinary ultrasound, and CT examination. Moreover, CT urography and cystoscopy were performed when needed.

The study endpoint was the overall survival time. Follow-up time was defined as the time from the postoperative period until the patient’s death or the last follow-up visit.

### Functional assessment

Three areas were included for the functional assessment: urinary control, sexual function, and complications. Functional follow-up of all patients was performed by a non-surgery-related trained physician and nursing team, either by direct questioning on return to the hospital for follow-up or by telephone. (1) Follow-up of urinary control function: Patients were asked about the number of routine pads used per day; time points were 3, 6, and 12 months postoperatively, allowing for a follow-up window with one-week redundancy before and after. Urinary incontinence was determined as follows (11–13): daily use of pads (≥ 1 pad) was considered incontinence, and complete dryness (0 pads) was considered urinary control recovery, i.e., no incontinence. (2) Sexual function follow-up: IIEF-5 score questionnaire (13, 14) and whether erectile dysfunction medication (mainly including type 5 phosphodiesterase inhibitors and herbal treatments such as Compound Xuanju capsule) was administered. The endpoint for sexual function follow-up was any time point occurring more than 6 months after surgery, at which point sexual function had returned to preoperative levels (e.g., IIEF-5 score ≥ 15). (3) Complications follow-up: Clavien classification was used, including voiding obstruction, urinary tract infection, re-hospitalization rates, and any other complications requiring therapeutic intervention (18).

### Statistical analysis

Statistical analysis was performed using the SPSS 26.0 software package. Normally distributed measurement data are presented as $\bar{\text{x}}$ *± s*, and comparisons between groups were conducted by Student’s t-test. Meanwhile, counting data were analyzed by *the χ* ^2^ test and Fisher’s exact test, and ranked data were analyzed by the Mann-Whitney rank-sum test. Caner-specific survival and overall survival were calculated by the Kaplan-Meier method, and the differences in survival curves between the two groups were tested by log-rank test. Differences were considered statistically significant at *p* < 0.05.

## Results

### Clinical baseline and perioperative characteristics of the two groups

**Figure 1:**
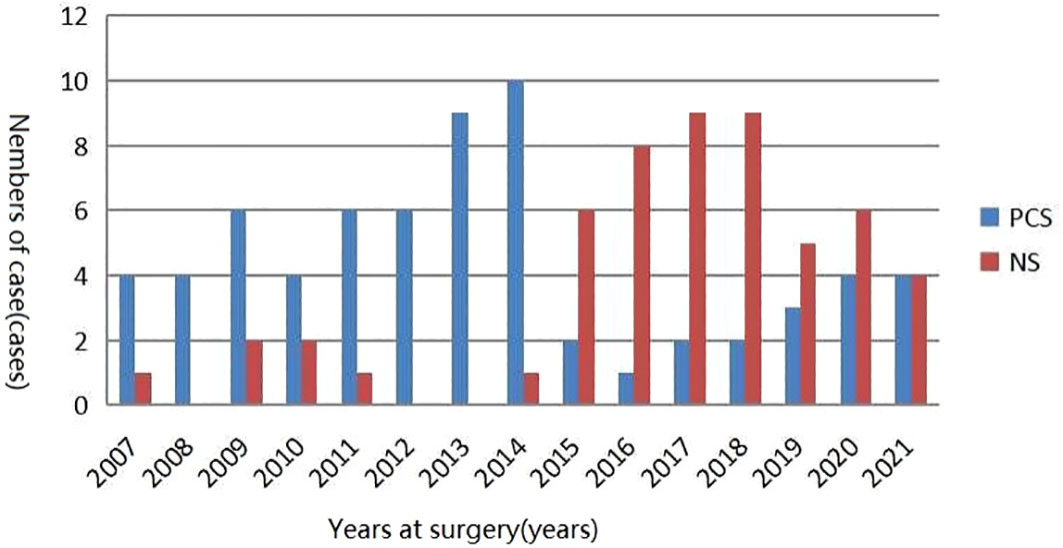
Distribution of PCS and NS surgeries performed in our surgical center over time. PCS, prostate capsule sparing; NS, nerve-sparing.

The clinical characteristics of patients in these two cohorts were presented in **Table 1**. A total of 67 and 54 patients underwent PCS and NS surgical treatments with a mean age of 67.79 and 66.72 years, respectively. The results revealed a non-significant difference between the two groups in terms of age, body mass index (BMI), tumor clinical stage, whether it was a high-grade tumor, tumor size, the use of neoadjuvant chemotherapy, and the time consumed for surgery (*p* > 0.05). Intraoperative blood loss and the number of lymph node removals were higher in the patients in the PCS group (*p* < 0.05), and the patients in the NS surgical group had a higher number of patients with preoperative hydronephrosis (*p* < 0.05). **Figure 1** illustrates the distribution of PCS and NS surgeries performed in our surgical center over times.

**Table 1:** Clinical baseline and perioperative characteristics of the PCS and NS groups.

Relevant factor	PCS (*n* = 67)	NS (*n* = 54)	*χ* ^2^/t	*p*
Age (years)	66.79 ± 9.34	66.72 ± 6.30	0.046	0.9
BMI (kg/m^2^)	22.98 ± 2.65	23.79 ± 2.30	-1.77	0.1
Clinical stage (cases, %)			0.625	0.7
<T1	15 (22.39)	9 (16.67)		
T2	42 (62.69)	36 (66.67)		
T3	10 (14.93)	9 (16.67)		
High-grade tumors (cases, %)	52 (77.61)	44 (81.48)	0.273	0.6
Tumor size (cm)	3.55 ± 9.34	3.35 ± 2.24	0.536	0.6
Preoperative hydronephrosis (cases, %)	3 (4.48)	11 (20.37)	/	0.009^*^
Neoadjuvant chemotherapy (cases, %)	11 (16.42)	17 (31.48)	3.815	0.1
Surgical time consumed (min)	335.90 ± 38.71	345.44 ± 45.31	-1.25	0.2
Blood loss (ml)	437.96 ± 163.14	357.06 ± 124.93	3.002	0.003
Number of lymph nodes removed	17.27 ± 4.29	15.31 ± 2.98	2.841	0.005
Neobladder (case, %)			0.370	0.9
Modified Studer’s ileum	56 (83.58)	47 (87.04)		
W-shaped ileum	5 (7.46)	3 (5.56)		
U-shaped ileum	4 (5.97)	3 (5.56)		
U-shaped sigmoid colon	2 (4.35)	1 (1.85)		

BMI, Body mass index; PCS, prostate capsule sparing; NS, nerve-sparing; ^*^Fisher’s exact test*; /, NO chi-square value*.

### Postoperative pathology and survival

**Figure 2:**
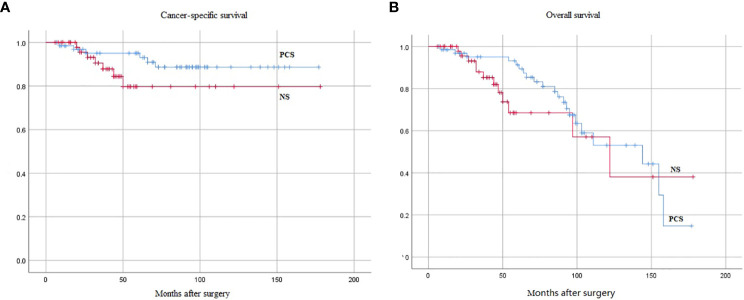
Kaplan-Meier curves of oncological endpoints between the two groups, the difference was not statistically significant (*p* > 0.05). **(A)** Cancer-specific survival (*p* = 0.123). **(B)** Overall survival (*p* = 0.224). PCS, prostate capsule sparing; NS, nerve-sparing.

Regarding postoperative pathological staging, 3 and 4 cases in the PCS and NS groups were at the T4 stage, while 19 and 16 cases had lymph node metastasis (N+), respectively. Postoperative adjuvant chemotherapy (*p* > 0.05) and radiotherapy [fewer patients received radiotherapy in the PCS group than in the NS group (5.97% vs. 18.52%, *p* = 0.032)] were administered to patients in both groups, taking into account postoperative pathological TNM (pTNM) staging, carcinoma *in situ*, surgical margins, or vascular infiltration or prostate cancer incidence. The results showed a non-significant difference between the two groups (*p* > 0.05). **Table 2** illustrates the pathologic features and oncological outcomes of PCS and NS surgeries. The median follow-up times were 144 and 122 months for PCS and NS, respectively. Cumulative survival estimated after surgery and cancer-specific survival were 93.0% and 88.7% at 5 and 10 years in the PCS group, as well as 79.7% and 79.6% (*p* = 0.123) in the NS group, respectively (**Figure 2A**). The 5-year and 10-year survival rates was 87.4% and 44.2% in the PCS group, as well as 68.5% and 38.0% (*p* = 0.224) in the NS group, respectively (**Figure 2B**). There was a non-significant difference between the two groups (*p* > 0.05) (**Figure 2**).

**Table 2:** Postoperative pathological characteristics of the PCS and NS groups.

Relevant factor (cases, %)	PCS (*n* = 67)	NS (*n* = 54)	*χ* ^2^	*p*
Pathologic stage			0.071	0.9
T1	25 (37.31	18 (33.33)		
T2	29 (43.28)	21 (38.89)		
T3	12 (17.91)	9 (16.67)		
T4	3 (4.48)	4 (7.41)		
Lymph node metastasis (N^+^)	19 (28.36)	16 (29.63)	0.024	0.9
Combined carcinoma in situ	7 (10.45)	2 (3.70)	/	0.2^*^
vascular infiltration	33 (49.25)	33 (61.11)	1.696	0.2
Prostate cancer	8 (11.94)	7 (12.96)	0.048	0.8
Positive surgical margin	3 (4.48)	1 (1.85)	/	0.4^*^
Adjuvant chemotherapy	10 (14.93)	13 (24.07)	1.626	0.2
Adjuvant radiotherapy	4 (5.97)	10 (18.52)	4.602	0.032

PCS, prostate capsule sparing; NS, nerve-sparing; ^*^Fisher’s exact test*; /, NO chi-square value*.

### Postoperative urinary control and sexual function recovery

Functional follow-up >12 months postoperatively was obtained in all patients. The rates of complete daytime urinary control (0 pads) at 3, 6, and 12 months postoperatively were 80.60%, 97.01%, and 100% in the PCS group and 53.70%, 85.19%, and 94.44% in the NS group (*p* = 0.002, 0.023, and 0.100, respectively). The PCS group was superior to the NS group (*p* < 0.05) in the early postoperative period (3 and 6 months), and the two groups gradually balanced by one-year postoperatively. Complete nocturnal urinary control (0 pads) at 3, 6, and 12 months postoperatively were 62.69%, 94.03%, and 98.51% in the PCS group and 40.74%, 72.22%, and 87.04% in the NS group (*p* = 0.016, 0.001, and 0.022, respectively), with the PCS group being superior to the NS group (*p* < 0.05). Recovery of erectile function showed that 51 and 39 patients in the PCS and NS groups requested treatment such as phosphodiesterase type 5 inhibitors or combined herbal medicine, respectively. Recovery to preoperative level (IEFF-5 score ≥ 15) was achieved in 42 (62.69%) and 22 (40.74%) patients in both groups (*p* = 0.016), respectively, and was significantly better in the PCS group than in the NS group. This advantage was more pronounced in patients who required pharmacologic intervention (*p* = 0.029), with a nonsignificant difference between the two groups without pharmacologic therapy (*p* = 0.700). **Table 3** summarizes the postoperative functional results.

**Table 3:** Results of postoperative functional recovery in PCS and NS groups.

Relevant factors (cases, %)	PCS (*n* = 67)	NS (*n* = 54)	*χ* ^2^	*p*
Complete daytime urinary control rate				
3 months	54 (80.60)	29 (53.70)	10.039	0.002
6 months	65 (97.01)	46 (85.19)	5.519	0.023
12 months	67 (100.0)	51 (94.44)	4.322	0.100
Nocturnal complete urinary control rate				
3 months	42 (62.69)	22 (40.74)	5.780	0.016
6 months	63 (94.03)	39 (72.22)	10.743	0.001
12 months	66 (98.51)	47 (87.04)	6.372	0.022
Erectile function restored to preoperative levels	42 (62.69)	22 (40.74)	5.780	0.016
untreated	9 (13.43)	6 (11.11)	0.148	0.700
medication	33 (49.25)	16 (29.63)	4.779	0.029

PCS, prostate capsule sparing; NS, nerve-sparing.

### Perioperative and long-term complications

During the postoperative perioperative period of 30 days, Clavien grade ≥ III complications occurred in three (incisional infected fissure 2 cases, intestinal obstruction 1 case) and four (incisional infected fissure, intestinal obstruction, intestinal fistula, and rectourethral fistula was 1 case, respectively) patients with PCS and NS procedures, respectively (*p* = 0.700). all of which were resolved after re-treatment. Complications such as urinary tract infections (pyelonephritis), adhesive bowel obstruction, and skin abscesses required re-hospitalization. In the PCS group, two patients (28-cm U-shaped and 36-cm W-shaped ileal neobladders 1 case, respectively) were experienced loss of bladder function (huge capacity and no contractility) at 11 and 14 years after surgery. The latter was also associated with multiple bladder stones, which required surgical treatments, such as cystostomy. The other was a patient with a 20-cm sigmoidal colon U-type neobladder who had three times bladder stones during five years after surgery, all of which required lithotripsy. Postoperative complications in both PCS and NS groups were shown in **Table 4**.

**Table 4:** Postoperative complications in both PCS and NS groups.

Relevant factors (cases, %)	PCS (*n* = 67)	NS (*n* = 54)	*χ* ^2^	*p*
Clavien ≥ III within 30 days	3 (4.48)	4 (7.69)	0.471	0.700
Re-hospitalization for urinary tract infections, etc.	14 (20.90)	10 (19.23)	0.106	0.700
Urinary retention requiring catheterization <6 months after surgery	11 (16.42)	2 (3.85)	/	0.036^*^
Intermittent catheterization required >6 months postoperatively	5 (7.46)	3 (5.77)	/	0.700^*^
Surgery required to treat bladder outlet obstruction	6 (8.96)	1 (1.92)	/	0.100^*^
Prostatitis/Epididymitis	12 (17.91)	1 (1.92)	/	0.006^*^
Loss of bladder function/recurrent bladder stones	3 (4.48)	0 (0.00)	/	0.300^*^

PCS, prostate capsule sparing; NS, nerve-sparing; ^*^Fisher’s exact test*; /, NO chi-square value*.

## Discussion

The ongoing debate about the oncological safety and functional efficacy of RC for bladder cancer with PCS is best addressed by randomized trials with NS. Our literature review found only one prospective randomized trial comparing PCS with NS. According to Jacob et al. (19), 40 patients were randomized to two groups, and survival and functional outcomes were assessed, revealing a nonsignificant difference between groups. Due to the small number of cases included, it was difficult to draw definitive conclusions. Herein, we report on patients who underwent PCS and NS surgery at our institution over 15 years and obtained long-term follow-up over one year or more, comparing the oncological and functional outcomes of the two surgical groups longitudinally.

Regarding surgical margins, Von Rundstedt et al. (20). have shown that the positive and negative predictive values of surgical margins for preoperative transurethral apical prostate and bladder neck biopsies were 6.9% and 99.5%, respectively, independent of intraoperative rapid frozen sections. The literature has reported that the rate of incidental prostate carcinoma in the NS preserved versus unpreserved was 38.5% and 42.5% (21), 39.1% and 52.2% (22), as well as 10% and 13.6% (23), respectively. Nonetheless, a nonsignificant difference was observed between the two groups. In our group, 12% and 13% of patients had incidental prostate adenocarcinoma, respectively. Moreover, in patients with positive surgical margins, no cases of death due to prostate cancer progression were identified in the follow-up.

Considering tumor recurrence and survival, in 2004, Botto et al. (24). have reported that distant metastasis incidence was 30% in patients treated with PCS. They speculated that transurethral endoscopic prostatectomy (TUR-P), performed a few days before RC, resulted in metastatic spread of the tumor through blood flow. Whether the higher distant metastasis incidence was due to Botto’s theory or poor patient selection, its incidence was low in this group, occurring in just one case. Recently, it has been demonstrated that the 5-year survival and caner-specific survival of NS total cystectomy was 71%–86.7% and 64%–86.7%, respectively (9). Furthermore, the 5-year survival of prostate-preserving versus non-preserving surgery was 70.6% and 74.2%, respectively, with a nonsignificant difference between the two groups (6). Caner-specific survival rates in the seminal vesicle prostate preserved versus non-preserved group were 73.41% and 80.65% (*p* = 0.165), with a nonsignificant difference between the two groups (25). The median follow-up time for PCS and NS in this group was 144 and 122 months, respectively. Caner-specific survival rates 5 and 10 years after surgery were 93.0% and 88.7% in the PCS group and 79.7% and 79.6% in the NS group, respectively. Meanwhile, the overall survival rates were 87.4% and 44.2% in the PCS group and 68.5% and 38.0% in the NS group, respectively. The results indicated a nonsignificant difference between the two groups (*p* > 0.05), and the two surgical styles did not affect the oncological results.

In terms of urinary control and sexual function, Gilbert et al. have stated that the quality of life of a reconstructed neobladder after any RC type was much lower than that of a native bladder on a functional level (26). There is controversy regarding the improvement in quality of life following different surgical approaches. Ziouziou I, et al. demonstrated that a better quality of life in urinary outcomes was found in patients with ileal conduit compared with orthotopic neobladder (27), while El Azab A, et al. showed that the quality of life of the neobladder was significantly higher than that of other urinary diversion modalities (14). In our clinical cohort, the majority of patients expressed satisfaction with both their quality of life and external appearance following the PCS and NS. According to most literature (5, 6, 9–11), when it comes to urinary control rates, NS procedures have shown daytime control rates ranging from 77% to 89.5% and nighttime control rates ranging from 54% to 88.9%. On the other hand, PCS procedures have demonstrated even higher daytime control rates, typically ranging from 94.4% to 100%, and nighttime control rates ranging from 70.2% to 96%. In addition, He et al. (23) found that in the NS group, the daytime urinary control rates at 3, 6, and 12 months postoperatively were 34.1%, 66.7%, and 90%, respectively, while the corresponding nighttime control rates were 27.3%, 57.1%, and 82.5%. For the PCS group, daytime control rates were 80%, 94.7%, and 92.9%, while nighttime control rates were 75%, 94.7%, and 92.9%. Furthermore, Wang et al. (24) reported that for NS procedures with an inter-fascial approach, daytime control rates were 36.8%, 52.6%, and 94.7%, and nighttime control rates were 5.3%, 21.1%, and 57.9%. For PCS procedures with an inter-fascial approach, daytime control rates were 76.9%, 84.6%, and 91.7%, while nighttime control rates were 46.2%, 58.3%, and 66.7%. Regarding the recovery of erectile function, NS procedures showed a range of 28.6% to 86.1% (6, 24, 25, 28), while PCS procedures demonstrated a range of 50% to 91.6% (6, 8, 9).

Consequently, we believe that postoperative urinary control lacks a universal consensus for a uniform standard in the literature. Various forms of questionnaires and urinary pad tests were used. For example, more urinary control was defined as using a maximum of one pad per day or night or as a criterion for urinary control based only on the subjective report of the patient. This results in the inability to compare postoperative urinary control more objectively. The questionnaire employed in our group included the more widely recognized complete urinary control and voiding pattern questionnaire, and the non-use of pads (0 pads) by the patient was deployed as a criterion for determining urinary control (11–13). For complete urinary control at 3, 6, and 12 months postoperatively, the NS group had daytime urinary control rates of 53.7%, 85.2%, and 94.4%, while the nighttime rates were 40.7%, 72.2%, and 87.04%. The PSC group had daytime urinary control rates of 80.8%, 97.0%, and 100%, whereas nighttime rates of 62.7%, 94.0%, and 98.5%. The PCS group was significantly better than the NS group (*p* < 0.05). For sexual function recovery determination, a preoperative baseline assessment of sexual function should also be performed to facilitate the selection of appropriate surgical methods. In our group, the IIEF-5 score was utilized for all patients with more active preoperative sexual function assessment (e.g., IIEF-5 score ≥ 15) and the requirement of preserving their sexual function. In our group, follow-up was investigated starting at six months postoperatively, and the time point when the sexual function returned to its best (e.g., IIEF-5 score ≥ 15 to preoperative level) was considered the endpoint. A total of 63% of PCS patients could return to the preoperative level, compared with 41% in the NS group, which was superior to that of the NS group (*p* < 0.05). Moreover, postoperative medication (phosphodiesterase type 5 inhibitor or combined with traditional Chinese medicines) significantly promoted the recovery of postoperative sexual function, consistent with that of a recent report (29).

Regarding postoperative complications, our results demonstrated a nonsignificant difference in Clavien grade ≥ III complications between patients undergoing PCS and NS surgeries during the 30-day perioperative period. Cases requiring hospitalization, such as urinary tract infections (pyelonephritis), were similar in both groups. Compared to NS group, dysuria (requiring intermittent clean catheterization) within six months after surgery was more frequent in the PCS group, with a nonsignificant difference in the long-term morbidity (> 6 months). A total of 9% of patients in the PCS group developed bladder neck obstruction (including reoccurring prostatic hyperplasia leading to dysuria) requiring surgical intervention, and 17.9% developed prostatitis or epididymitis. Additionally, the neobladder (28-cm ileal U-type and 36-cm ileal W-type) loss of function (huge capacity and no contractility) occurred in two cases, and bladder stones (20-cm sigmoidal U-type neobladder) occurred (three times) in one case within five years after surgery. All three patients had dysuria symptoms, but cystoscopy showed no bladder neck obstruction, such as prostatic hyperplasia. These complications may be attributed to factors such as the choice of bowel material and neobladder morphology used in the construction. Since all of these complications occurred in the PCS group, and more patients had difficulty urinating within six months after the operation, we hypothesize that whether the PCS technique is more prone to potential occult vesicourethral obstruction needs further exploration. It can be indicated that long-term close follow-up of patients with the PCS technique is more important.

In terms of insufficient research, this study is an empirical summary of a larger number of cases and reports on the oncological and functional outcomes as well as complications of PCS and NS techniques. However, all the patients who received RC in this group were highly selected and only from a retrospective analysis of a single center. The selection of the two surgical modalities was non-randomized, and their comparison may be subjected to a high degree of bias. Our main purpose was to report the oncological and functional outcomes of two different preserved nerve radical cystectomy.

## Conclusion

The PCS provides better and faster recovery of urinary control and sexual function than the NS technique, as it does not affect the oncological outcomes. Nevertheless, PCS is susceptible to bladder-neck obstruction complications and requires close long-term follow-up.

## Data availability statement

The original contributions presented in the study are included in the article/supplementary material. Further inquiries can be directed to the corresponding author.

## Ethics statement

The studies involving humans were approved by Jinhua Hospital Affiliated to Zhejiang University School of Medicine (Jinhua Central Hospital), Jinhua, China, 321000. The studies were conducted in accordance with the local legislation and institutional requirements. Written informed consent for participation was not required from the participants or the participants’ legal guardians/next of kin in accordance with the national legislation and institutional requirements.

## Author contributions

ZZ: Writing – original draft, Writing – review & editing. YZ: Writing – original draft, Writing – review & editing. HS: Writing – original draft. PZ: Writing – original draft. YX: Writing – original draft. SH: Writing – original draft.
